# Anticancer Activity of *Smallanthus sonchifolius* Methanol Extract against Human Hepatocellular Carcinoma Cells

**DOI:** 10.3390/molecules24173054

**Published:** 2019-08-22

**Authors:** Phyu Phyu Myint, Thien T. P. Dao, Yeong Shik Kim

**Affiliations:** 1 Department of Chemistry, Loikaw University, Loikaw 09013, Myanmar; 2 College of Pharmacy and Natural Products Research Institute, Seoul National University, Seoul 08826, Korea

**Keywords:** *S. sonchifolius* leaf, HepG2 cells, MTT assay, cell cycle arrest, anti-liver cancer drug, antioxidant

## Abstract

**Background:** This research aimed to investigate the cytotoxicity of methanol extract of *Smallanthus sonchifolius* leaf (YLE) against a human hepatocellular carcinoma cell line (HepG2). This plant is currently used as a traditional herbal remedy in the treatment of liver diseases in some rural parts of Myanmar. **Methods**: The cytotoxic activity of the plant extract against the cancerous cell line was assessed using an MTT assay. YLE demonstrated a significant effect (IC50 = 58.2 ± 1.9 μg/mL) on anti-cancer activity, which was further investigated using various assays including an in vitro cell migration assay, a colony formation assay, cell cycle analysis, western blot analysis, and a ROS assay. The significance of the phytochemical constituents of YLE could be identified using LC/Q-TOF-MS techniques. **Results**: We putatively identified the active components in YLE, which were possibly melampolide-type sesquiterpenoids. YLE showed an inhibitory effect on HepG2 cell proliferation and cell migration. YLE also induced cell cycle arrest and necrosis in a dose-dependent manner. Additionally, YLE significantly suppressed ROS formation in HepG2 cells. **Conclusions**: These findings suggest that YLE is sufficient for application as a promising anti-liver drug in herbal medicine.

## 1. Introduction

In 2018, liver cancer was the sixth most common cancer and the fourth leading cause of cancer deaths worldwide [1]. The highest incidence of this cancer can be seen in East Asia, Southeast Asia, and North and Southern Africa [2]. Based on the database of the International Agency for Research on Cancer (IARC), there were more than 69,000 new cancer cases in Myanmar in 2018 and liver cancers were in the top 5 in terms of incidence, mortality, and prevalence by cancer site [1]. Currently, the Ministry of Health and Sports from Myanmar supports the implementation of the National Cancer Control Plan, focusing on priority activities and maximizing efforts in line with the respective mandates, priorities, and areas of expertise of the partner and to achieve better results for cancer prevention, care, and control.

Testing, annual screenings, and early intervention for cancers are currently inadequate on many accounts, which include the rise in population, an inadequate supply of drugs, the cost of treatments, the side effects of several synthetic medicines, and increasing resistance to the drugs used. In most rural areas, herbal medicine has been used for decades by traditional practitioners to treat cancer problems. Medicinal plants have long been used in the treatment of liver diseases or the maintenance of a healthy liver. Yacon, or *Smallanthus sonchifolius* ((Poepp. & Endl.) H. Rob.), is a plant belonging to the Asteraceae family, native to the Andean regions of South America [3]. The plant contents include phenolic acids, flavonoids, and sesquiterpene lactones [4,5]. Yacon has been used as a functional food with multiple beneficial effects on the body, including as an antimicrobial, as an antioxidant, hypolipidemic effects, and probiotic substances [3,6]. The plant was cultivated in Myanmar in the 2000s. It has become increasingly popular as medicated green tea for diabetes patients and its use is wide-spread.

In recent years, Yacon has emerged as a potential anti-cancer agent. Previous in vitro studies indicated that the crude extract of Yacon and the phytochemicals derived from the plants exerted the cytotoxicity against breast cancer [7], colon cancer [7,8], and cervical cancer [9,10]. The anticancer property was attributed to sesquiterpene lactones in Yacon [9,10,11]. In addition, Yacon has been well-known to have antioxidant effects because of an abundant amount of polyphenols, which are found at high quantities in leaves or stems of the plant [6]. Recent studies have indicated that antioxidants might possess anti-tumor and hepatoprotective effects, although the mechanism needs further investigation [12].

This research aimed to evaluate the effects of Yacon leaf extract (YLE) on liver cancer in vitro using hepatocellular carcinoma HepG2 cell line, which is the most commonly used in drug metabolism and hepatotoxicity studies. HepG2 cells are nontumorigenic with high proliferation rates and an epithelial-like morphology that performs many differentiated hepatic functions [13]. The medicinal plant is of high pharmacological importance, but it is still not reported for its chemotherapeutic potential as an alternative medicine for liver cancer disease. Our results may provide scientific evidence for the therapeutic potential of this plant, as a functional food, on liver cancer.

## 2. Results

### 2.1. Cytotoxicity of YLE by MTT Assay

**Figure 1:**
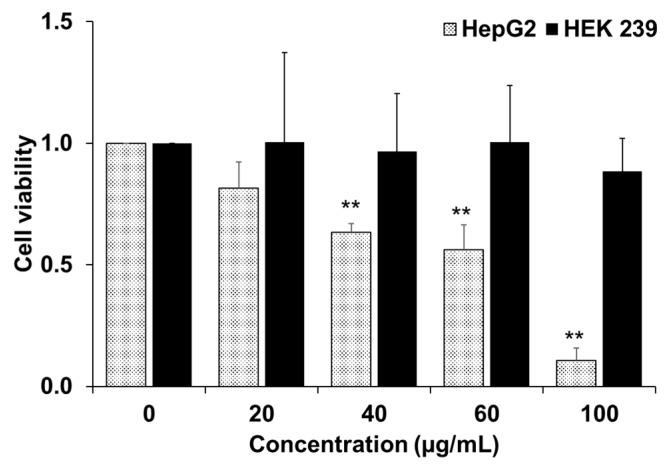
Cell viability of HepG2 and HEK 239 cells after being treated with different concentration of YLE. Data are presented as means ± standard deviation (S.D) (*n* = 3); ** *p* < 0.01 vs. control group.

The sample was evaluated for cytotoxic activity on human hepatoma carcinoma cell lines (HepG2), as presented in Figure 1. The results of the MTT assay showed a dose-dependent reduction in cell viability of HepG2 cells while YLE did not affect those of non-tumor HEK 239 cells after 24 h treatment. The calculated IC_50_ of YLE on HepG2 was 58.2 ± 1.9 µg/mL.

### 2.2. YLE Reduces Colony Formation of HepG2 Cells

**Figure 2:**
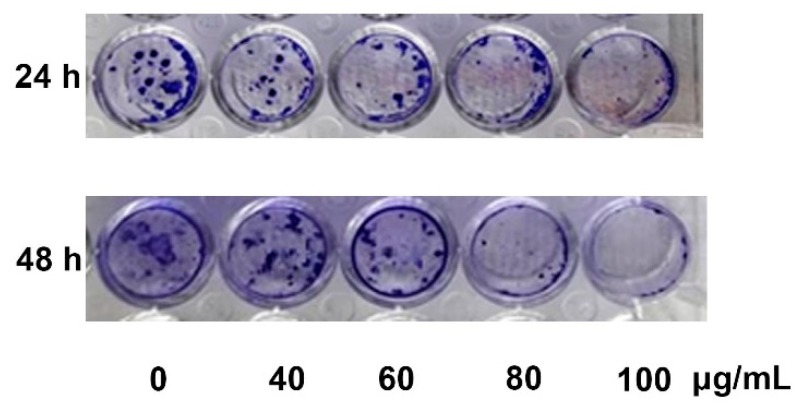
HepG2 cells after 24 h and 48 h treatment with YLE were allowed to grow into visible colonies for an additional 2 weeks. The figure presents one of three independent experiments.

To determine the effect of YLE on the replicative potential and the longer-term viability of liver cancer cells under colony-forming culture conditions, we treated HepG2 cells with various concentrations of the extracts for 24 h or 48 h, then conducted a crystal violet-based clonogenic assay. Data showed that YLE significantly inhibited colony formation of HepG2 after 14 days. We found that cell proliferation rates gradually decreased as the concentration of the extract was increased. These findings suggest that YLE exerts its strong inhibitory effect on longer-term viability liver cancer cells in a dose-dependent manner. The full inhibitory effect of YLE on the HepG2 clonogenicity could be observed within 24 h at 100 μg/mL dose and 48 h at 80 and 100 μg/mL (Figure 2).

### 2.3. Effect of YLE on Wound Healing in HepG2 Cells

**Figure 3:**
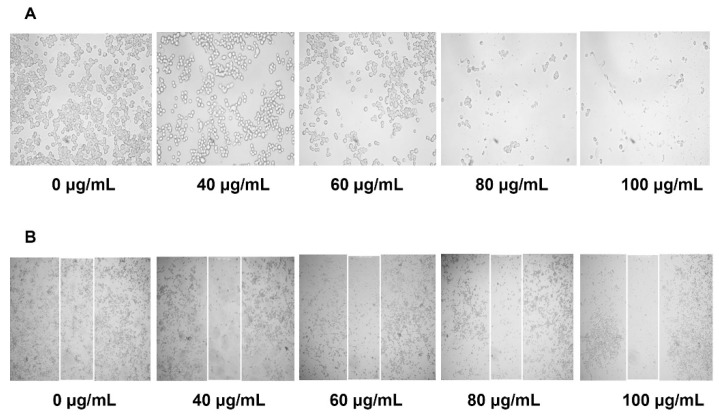
Effect of YLE on (**A**) cell morphology and (**B**) migration ability of HepG2 cells. The figures show one of three independent experiments.

Migration of cells plays a vital role in cancer cells survival; thus, we conducted a wound-healing assay to examine the effect of YLE on the healing process in HepG2 cells. In this study, at the start of in vitro scratch test, there were little or no cells inside the scratch region. After 24 h exposure to YLE, it was observed that the cell migrated towards the induced gap (Figure 3). The control sample migration (0 μg/mL) was noted to be the highest in all the cell lines tested. YLE significantly decreased the migration in HepG2 cells.

### 2.4. Effect of YLE on ROS Production

**Figure 4:**
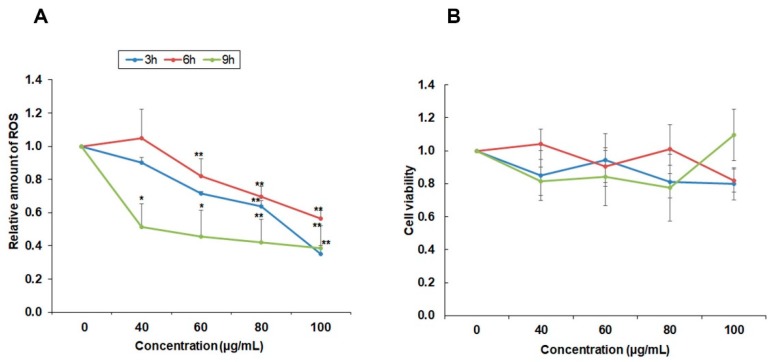
Effect of YLE on ROS formation of HepG2 cells. (**A**) Antioxidant effect of YLE on HepG2 cells after 3 h, 6 h, and 9 h. Data are expressed as averages ± S.D (*n* = 3); * *p* < 0.05, ** *p* < 0.01 vs. control group. (**B**) Effect of YLE on cell viability. Data indicated no specific change between treatment groups and the control group after indicated times (3 h, 6 h, 9 h).

To evaluate the influence of YLE on mediating ROS generation in HepG2 cells, we incubated cells with different concentrations of YLE in indicated times (3 h, 6 h, 9 h). The intracellular ROS was measured via 2′,7′-dichlorofluorescein diacetate (DCF-DA) fluorescence. As displayed in Figure 4, intracellular ROS levels were significantly decreased in a concentration-dependent manner (Figure 4A), while the cell viability of HepG2 was not affected in short-time treatment (Figure 4B). These data proved that a reduction of ROS production in HepG2 cells was not due to decreasing numbers of living cells. The ROS-inhibitory effect of YLE showed similar patterns after 3 h and 6 h of treatment. The specific changes could be observed only at high concentrations, such as 80 and 60 μg/mL, respectively. However, after 9 h of treatment, the ROS production was inhibited notably (>50%) even at the lowest concentration (40 μg/mL) used in the experiments. These results suggest that the antioxidant effect of YLE possibly led to induced cytostasis in HepG2 cells after 24 h treatment.

### 2.5. YLE Induces Cell Cycle Arrest in HepG2 Cells

**Figure 5:**
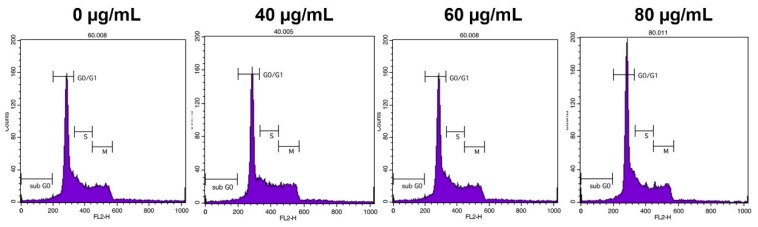
Effect of YLE on the cell cycle of HepG2 cells. The figure represents triplicate experiments.

Next, we examined the effect of YLE on the cell cycle of a HepG2 liver cancer cell line after 24 h treatment using flow cytometry. Figure 5 shows the relative percentages of HepG2 cells in each phase of the cell cycle following treatment. There was a dose-dependent increase in the percentage of cells in the G0/G1 phase. The data in this study suggested that YLE could inhibit cell proliferation of HepG2 cells by inducing the cell cycle at the G0/G1 phase.

### 2.6. YLE Induces Necrosis in HepG2 Cells

**Figure 6:**
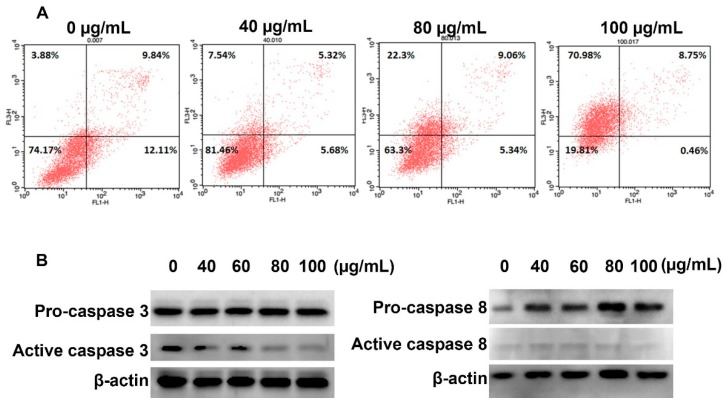
Effect of YLE on (**A**) the cell death mechanism and (**B**) the expression levels of proteins in HepG2 cells. The figure shows one of three independent experiments.

To clarify the mechanism of YLE on inducing cell death in HepG2 cells, we stained the YLE-treated cells with Annexin V/PI after 24 h exposure and measured the fluorescence by flow cytometry. The percentage of each subpopulation of cells is described in Figure 6. We found that YLE induced the loss of cell membrane integrity, which was indicated by the increase in numbers of PI-positive cells. The proportion of only the PI-positive cell population treated with 100 µg/mL YLE was approximately 20-fold higher than the one in the control group. Additionally, YLE did not trigger caspase activation or cleavage of caspase proteins, such as caspase 3 and caspase 8. These data suggest a necrotic mode of cell death induced by YLE.

### 2.7. Metabolites Identification of Methanol Extract of YLE

**Figure 7:**
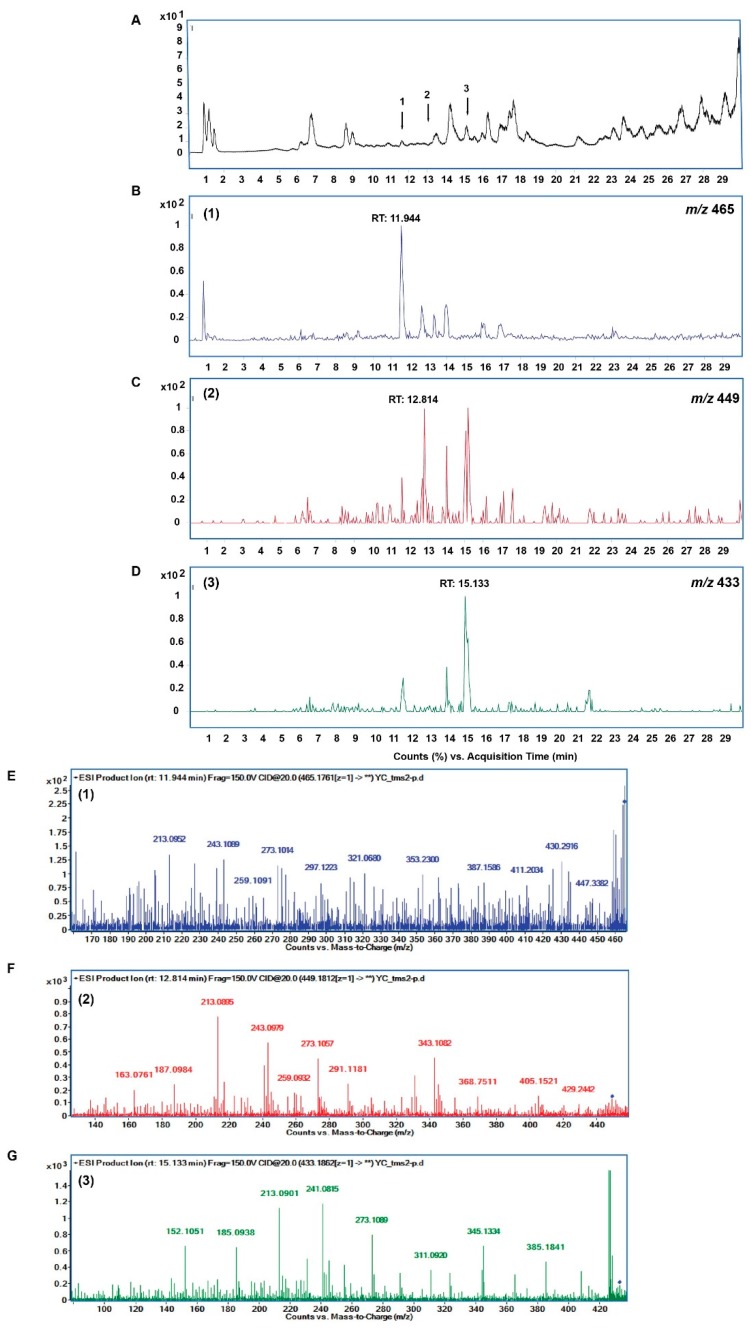
TIC in the positive mode of YLE (**A**). EIC of *m/z* 465.1761 (±10.0 ppm) [C_23_H_28_O_10_ + H]^+^ (**B**); EIC of *m/z* 449.1812 (±10.0 ppm) [C_23_H_28_O_9_ + H]^+^ (**C**); EIC of *m/z* 433.1862 (±10.0 ppm) [C_23_H_28_O_8_ +H]^+^ (**D**). Product-ion chromatograms of compound 1 (**E**), compound 2 (**F**), and compound 3 (**G**).

In this study, we confirmed the presence of several metabolites in YLE based on the characteristic fragments in the MS spectra previously described in the literature [5]. We focused on raw formulas of several melampolide sesquiterpene lactones, which were previously described as main components in Yacon, as follows: C_23_H_28_O_8_, C_23_H_28_O_9_, and C_23_H_28_O_10_ [5]. Figure 7 displays the total ion current chromatogram (TIC) of the extract (Figure 7A) and the obtained extracted-ion chromatograms (EIC) showing the series of peaks with *m/z* values corresponding to the three selected molecular formulas (Figure 7B–D). Compound 1 (retention time (rt) 11.94), compound 2 (rt. 12.81 min), and compound 3 (rt.15.13) exhibited a protonated molecular ion [M + H]^+^ at *m/z* 465.1657 [C_23_H_28_O_10_ + H]^+^, *m*/*z* 449.1812 [C_23_H_28_O_9_ + H]^+^, and *m*/*z* 433.1788 [C_23_H_28_O_8_ + H]^+^, respectively. These formulas shared the common product-ions that were characterized for uvedalin moiety with *m*/*z* 213.0901; 241.0815; 273.1809, putatively identified as an isoform to enhydrin (C_23_H_28_O_10_), uvedalin (C_23_H_28_O_9_), and polymatin B (C_23_H_28_O_8_) [5] (Figure 7E–G).

## 3. Discussion

Yacon (*S. sonchifolius*), a common edible plant grown throughout the world, is well known for its anti-diabetic properties [14]. It is also demonstrated to have several other pharmacological properties, including anti-inflammatory, anti-oxidant, anti-allergic, and anti-cancer effects [15]. The cytotoxicity potential of hexane, methanol, and dichloromethane extracts of Yacon leaves was assessed against MCF-7 and HT-29 cell lines by using the AlamarBlue^®^ assay [7]. Sesquiterpene lactones from Yacon leaves, such as enhydrin, uvedalin, and their derivatives, also exhibited cytotoxic activity against MGC80-3 [16], HeLa, HL-60, and B16-F10 cell lines [9]. However, there has been no report yet to evaluate the anti-liver cancer activity of *S. sonchifolius* on liver cancer cells. Therefore, this study aimed to investigate the anti-cancer effect of YLE on HepG2 cells in terms of inhibiting cell proliferation and migration. In addition, we also examined the YLE effect on cell cycle and ROS generation in this cell line.

In the current study, YLE was found to show a potent inhibitory effect on HepG2 cell survival. Firstly, we examined the toxicity of YLE on liver cancer HepG2 cells. The calculated IC_50_ (58.2 ± 1.9 µg/mL) implied the promising inhibitory effect against these cancer cells. Indeed, the colony formation of HepG2 cells was significantly suppressed with increasing concentration of YLE up to more than 90% at 100 µg/mL, in comparison with the control, after 24 h or 48 h treatment. Taken together, these results suggested that YLE demonstrates a long-term suppressive effect on cell proliferation of HepG2 in a concentration-dependent manner.

It is known that metastasis is one of the leading causes of death from cancer and it is complicated to examine [17]. During tumor metastasis, malignant cells migrate into neighboring healthy tissues, contributing to tumor development. In this study, YLE could effectively prevent the migration of HepG2 in a dose-dependent manner. Therefore, YLE could contribute to hinder metastasis progression in hepatocellular carcinoma.

To control cancer growth, inhibition of the progression of the cell cycle is one of the essential strategies [18]. In this study, flow cytometry analysis demonstrated that the extract dose-dependently increased the percentage of cells of the G0/G1 phase of the HepG2 cell cycle. This observation reveals that YLE induces cell cycle arrest at the G0/G1 phase of the cell cycle.

In term of inducing cancer cell death, we found that YLS induced necrosis in HepG2 cells. The number of cells in the necrosis subpopulation significantly increased from 3.88% (control group) to 70.98% (100 μg/mL YLE-treatment group), whereas YLE did not activate caspase 3 or caspase 8, which regulate apoptosis in cells.

High levels of ROS in cancer cells have been found in almost all cancer cells due to high metabolomic activity and they have specific functions in cancer cell development [19]. ROS have been reported to be involved in cell proliferation, cell survival, cell cycle progression, and angiogenesis. Therefore, suppressing ROS may be a useful strategy in cancer treatment [20]. Since YLE has been reported to contain a large number of polyphenols [4], we sought to evaluate the antioxidant effect of YLE on HepG2 cells. YLE could notably reduce ROS in HepG2 in both dose and time-dependent manners. These observations may support the inhibitory effect of YLE on HepG2 cell proliferation.

The presence of sesquiterpene lactones, such as polymatin B, enhydrin, and uvedalin, was also confirmed by HPLC coupled with high-resolution mass tandem analysis [5]. These compounds have been demonstrated to have cytotoxicity as well as to induce apoptosis or necrosis in several cancer cell lines [11]. In the current study, we partly confirmed the presence of these sesquiterpenoids sharing the common fragment characteristic of the uvedalin moiety. These compounds may be significant as chemical defenses for human hepatocellular carcinoma HepG2 cells. Although the effect of individual constituents of this plant extract on HepG2 cells needs to be further investigated, the findings in the current study suggest that *S. sonchifolius* leaf could be recommended as a potential source of a chemopreventative agent against liver cancer.

## 4. Materials and Methods

### 4.1. Preparation of the Samples

The Yacon (*Smallanthus sonchifolius* ((Poepp. & Endl.) H. Rob.) leaves were collected from Pindaya Township, Shan State, in the eastern part of Myanmar. The sample was identified at the Department of Botany, University of Yangon. The leaves were cleaned, carefully dried in shadow, and powdered. About 2 g of ground powder was soaked in 20 mL methanol and shaken for 8 h at 180 rpm and 37 °C. The soaked substance was filtered throughout 110 mm filter paper (Hyundai, Seoul, South Korea). The solvent was eliminated with a rotary evaporator (SB-1200, EYELA, Shanghai, China) at 60 °C. The residue was placed in a freeze drier (Operon, Korea) to dry. The crude extract was kept in a refrigerator at 4 °C and protected from light. The experiment was performed in triplicate. Four-hundred mg of the sample extract was dissolved in 1 mL of dimethyl sulfoxide (DMSO) and then serially diluted to 100, 60, 40, and 20 μg/mL for further biological experiments. All other chemical reagents were from Sigma-Aldrich Chemical Company (St. Louis, MO, USA) unless otherwise noted.

### 4.2. Cell Line and Culture Medium

HepG2 and HEK 239 cell lines were obtained from the American Type Culture Collection (ATCC). Cells were cultured in Dulbecco’s Modified Eagle Medium (DMEM) media supplemented with 10% (*v/v*) fetal bovine serum and 1% (*v/v*), 100 U/mL penicillin and 100 μg/mL streptomycin. Cells were cultured in an incubator at 37 °C and 5% CO_2_ humidified atmosphere. Media were changed every 2 to 3 days. Reagents and media for cell culture were purchased from GenDePOT (Katy, TX, USA).

### 4.3. Cytotoxicity Assay (MTT Assay)

The cellular toxicity of the YLE on cultured cells was measured using 3-(4,5-dimethylthiazol-2-yl)-2, 5-diphenyl tetrazolium bromide (MTT). Cells were grown in a 96-well plate at a density of 1 × 10^4^ cells per well. The cells are allowed to grow overnight in a cell culture incubator. Then, we treated cells with different concentrations of samples (0, 20, 40, 60, and 100 μg/mL) for 24 h. Later, cells were washed twice with phosphate buffer saline (PBS). MTT solution was added to each well (final concentration of MTT was 0.5 mg/mL) and the plate was incubated for 3 h. Finally, the medium was replaced by DMSO to solubilize the formazan crystals. The optical density was measured by absorbance at 595 nm with a microplate spectrophotometer (SpectraMax 190, Molecular Devices, San Jose, CA, USA). We identified the IC_50_ value using the ED50 Plus v1.0 online software (National Institute of Respiratory Diseases (INER), Mexico) as described elsewhere [21].

### 4.4. Cell Colony Formation Assay

Cells were seeded at a density of 1 × 10^3^ cells/well in 6-well plates and allowed to attach overnight. Then, the cells were treated with YLE (0, 40, 60, 80, and 100 μg/mL) for 24 h or 48 h. After treatment, cells were continuously grown for another 2 weeks. The media were changed every 3 days. After 14 days, cells were washed with PBS and fixed with 3.7% formaldehyde solution for 30 min before being stained with 0.5% crystal violet for another 30 min at room temperature (RT). Cells were washed with water to remove the dye and then photographed.

### 4.5. Cell Migration Assay (Wound–Healing Assay)

We seeded cells in a 24-well plate (5 × 10^4^ cells/well) and cultured at 37 °C, 5% CO_2_ for 24 h. The 80–90% cell confluence was observed before the scratch assay was performed. We stimulated a wound using a sterile 200 μL pipette tip. After washing with PBS to remove loosened debris, cells were treated with YLE (0, 40, 60, 80, and 100 μg/mL) for 24 h at 37 °C, 5% CO_2_. After treatment, cells were washed twice with PBS. The images of cells were captured under a CKX41 microscope (Olympus, Japan) at 400× magnification. Images were processed with ProgRes Capture Pro software v.2.8.8 (JENOPTIK Optical Systems, Jena, Germany).

### 4.6. Reactive Oxygen Species (ROS) Production Assay

The level of intracellular ROS was determined by the change in fluorescence resulting from the oxidation of the fluorescent probe dichlorofluorescein diacetate (DCF-DA). We seeded cells at a density of 1 × 10^6^ cells/well in 6 wells plate and then treated them with 0, 40, 60, 80, and 100 ug/mL of YLE for 3, 6, and 9 h. After indicated periods, cells were washed twice with DPBS and stained with dichlorofluorescein diacetate (DCF-DA, 10 μM) at 37 °C for 30 min in the dark. After washing twice with PBS, the fluorescence generation was measured in a microplate reader, (excitation wavelength (ext.): 485 nm; emission wavelength (emi.): 535 nm). Data were expressed as the percentage of ROS relative to untreated control groups.

### 4.7. Cell Cycle Analysis

HepG2 cells at a concentration of 1 × 10^6^/well cells were cultured in 6-well plates and treated with different concentrations of YLE for 24h. After treatment, the cells were washed with PBS and harvested using a centrifuge at 1300 rpm for 3 min. Then, cells were re-suspended and fixed with 70% ethanol at −20 °C for 1 week. After fixing, we harvested cells by washing with PBS and centrifuging at 2000 rpm for 3 min. Cells were incubated with RNase (200 μg/mL) at 37 °C for 30 min to remove cellular RNA, then stained with propidium iodide (PI, 100 μg/mL) for another 10 min at RT in the dark. Finally, the cells were analyzed by flow cytometry (BD FACSCalibur, BD Biosciences, USA) according to detected signals in the FL2 channel (ext: 488 nm, emi: 564–606 nm) while data were analyzed with Cell Quest Pro software (BD Biosciences, San Jose, CA, USA).

### 4.8. Annexin V/PI Assay

HepG2 cells were seeded in the 6-well plates (1 × 10^6^ cells/well). After overnight incubation, we treated cells with indicated concentrations of YLE for 24 h. Cells were washed with PBS and harvested using a centrifuge at 2000 rpm for 3 min for the following analysis. To distinguish apoptotic and necrotic cell death in HepG2 cells, we used the BD Annexin V: FITC Apoptosis Detection Kit I (BD Biosciences, San Diego, CA, USA), according to the manufacture’s direction. The cells were analyzed by flow cytometry (BD FACSCalibur, San Jose, CA). Signals were detected in the FL1 channel (for Annexin V) and FL3 channel (for PI), while data were analyzed with Cell Quest Pro software.

### 4.9. Western Blotting

The HepG2 cells were seeded in a 6-well plate at 1 × 10^6^ cells/well and treated with various concentrations of YLE for 24 h. The whole cells lysates were prepared by homogenizing cells in a prepared lysis buffer (20 mM HEPES (pH 7.6), 350 mM NaCl, 20% glycerol, 0.5 mM EDTA, 0.1 mM EGTA, 1% NP-40, 50 mM NaF, 0.1 mM DTT, 0.1 mM PMSF, and a protease inhibitor cocktail) on ice for 30 min, followed by collecting the supernatant using a centrifuge at 15,000 rpm for 10 min. The concentration of protein was identified using a Bradford assay. The equal amounts of proteins were separated by electrophoresis on 10% SDS gels and transferred to nitrocellulose membranes. Blots were blocked with 5% BSA for 1 h prior to incubating with the following primary antibodies: β-actin conjugated with HRP (Santa Cruz, 47778) as1:3000, pro-caspase 3 (rabbit, Santa Cruz H-277: sc-7148) as 1:1000, cleaved caspase 3 (rabbit, Abcam ab2302) as 1:1000, pro-caspase 8 (rabbit, Santa Cruz, sc-6134) as 1:1000, and cleaved caspase 8 (rabbit, Cell Signaling Technology, 9496) as 1:1000, at 4 °C overnight. After washing the membrane and incubating with goat anti-rabbit IgG (H + L) secondary antibody conjugated with HRP (GeneTex, GTX213110, 1:3000) for 1 h at RT, the bands of interest were detected using an EZWestern ECL kit (Daeillab Service, Seoul, South Korea) and then photographed on the LAS-4000 imaging system (GE Healthcare, Chicago, IL, USA).

### 4.10. LC-Q-TOF-MS Analysis of YLE

Since the cytotoxicity of YLE was attributed to its active ingredients, which were known to be sesquiterpenoids, we confirmed the presence of these compounds using liquid chromatography analysis in an HPLC system (Agilent Technologies, Santa Clara, CA, USA) linked to a G6530A ESI-Q-TOF MS spectrometer (Agilent Technologies). Chromatographic separations were conducted using a C18 column (2.1 × 150 mm, 3.5 μm, Agilent Technologies). The mobile phases were water with 0.1% formic acid (A) and acetonitrile with 0.1% formic acid (B). The gradient program consisted of the following: 0–15 min, 5–50% B; 15–20 min, 50% B; 20–30 min, 50–95% B; followed by 95% B for 10 min washing. The flow rate was 0.3 mL/min and the temperature was kept at 40 °C. The injection volume was 3 μL. The instrument was operated with an ESI source in positive ion mode. The mass determination was performed using the following MS conditions: Ion spray voltage of 400 kV; desolvation temperature, 350 °C; desolvation gas flow rate, 10 L/min. The fragmentor was set at 150 V. The full-scan mass spectra were acquired within an *m/z* range of 100 to 1200 *m/z* in the MS mode followed by the target MS/MS mode. Data acquisition and proceeding were performed using Mass Hunter Qualitative Analysis software (Agilent Technologies). The compounds were putatively identified based on their mass tandems in comparison with published data.

### 4.11. Statistical Analysis

In this study, we performed all experiments in triplicate and analyzed results using one-way ANOVA followed by a Dunnett’s test (SPSS version 25.0; Chicago, IL, USA). Probabilities of *p* < 0.05 were considered significant.

## 5. Conclusions

In this study, we partially identified the active ingredients present in the YLE, which are possibly melampolide sesquiterpene lactones with uvedalin moiety. The results in this study indicate that YLE appears to be capable of killing malignant liver cancer cells by inhibiting the growth and migration in addition to inducing necrosis and cell cycle arrest. Furthermore, we also confirmed the antioxidant effect of YLE on liver cancer cells. To conclude, these findings suggest *S. sonchifolius* (Yacon) is a promising potential anti-liver cancer agent in the area of herbal medicine. Further research regarding the role of each active compound in YLE towards anti-liver cancer activity would be worthwhile.
